# Virulence Characteristics and an Action Mode of Antibiotic Resistance in Multidrug-Resistant *Pseudomonas aeruginosa*

**DOI:** 10.1038/s41598-018-37422-9

**Published:** 2019-01-24

**Authors:** Wontae Hwang, Sang Sun Yoon

**Affiliations:** 1 Department of Microbiology and Immunology, Brain Korea 21 PLUS Project for Medical Sciences, Seoul, Korea; 2 Institute for Immunology and Immunological Diseases, Yonsei University College of Medicine, Seoul, 03722 Korea

## Abstract

*Pseudomonas aeruginosa* displays intrinsic resistance to many antibiotics and known to acquire actively genetic mutations for further resistance. In this study, we attempted to understand genomic and transcriptomic landscapes of *P*. *aeruginosa* clinical isolates that are highly resistant to multiple antibiotics. We also aimed to reveal a mode of antibiotic resistance by elucidating transcriptional response of genes conferring antibiotic resistance. To this end, we sequenced the whole genomes and profiled genome-wide RNA transcripts of three different multi-drug resistant (MDR) clinical isolates that are phylogenetically distant from one another. Multi-layered genome comparisons with genomes of antibiotic-susceptible *P*. *aeruginosa* strains and 70 other antibiotic-resistance strains revealed both well-characterized conserved gene mutations and distinct distribution of antibiotic-resistant genes (ARGs) among strains. Transcriptions of genes involved in quorum sensing and type VI secretion systems were invariably downregulated in the MDR strains. Virulence-associated phenotypes were further examined and results indicate that our MDR strains are clearly avirulent. Transcriptions of 64 genes, logically selected to be related with antibiotic resistance in MDR strains, were active under normal growth conditions and remained unchanged during antibiotic treatment. These results propose that antibiotic resistance is achieved by a “constitutive” response scheme, where ARGs are actively expressed even in the absence of antibiotic stress, rather than a “reactive” response. Bacterial responses explored at the transcriptomic level in conjunction with their genome repertoires provided novel insights into (i) the virulence-associated phenotypes and (ii) a mode of antibiotic resistance in MDR *P*. *aeruginosa* strains.

## Introduction

Antibiotic-resistant infections are now a serious problem worldwide. In 2017, a strain of *Klebsiella pneumoniae* that became resistant to all available antibiotics caused a fatal infection in the US^1^. Globally, more than 0.7 million people die each year from resistant infections and it was estimated that 10 million people will die from antimicrobial-resistant (AMR) infections in 2050^2^. There is a famous collection called ESKAPE (*Enterococcus faecium*, *Staphylococcus aureus*, *Klebsiella pneumonia*, *Acinetobacter baumannii*, *Pseudomonas aeruginosa*, and *Enterobacter* species) composed of troublemaker pathogens that easily acquire antibiotic resistance^3^. In the US, the Centers for Disease Control and Prevention reported that 51,000 patients became infected with *P*. *aeruginosa* annually and 13% among those are infected by multidrug-resistant (MDR) strains^4^.

*P*. *aeruginosa* strains contain genomes of approximately 5 to 7 Mbp, and significant numbers of their conserved genes encode regulatory proteins. This suggests that *P*. *aeruginosa* are capable of responding to various environmental stresses^5^. In addition, *P*. *aeruginosa* has intrinsic antibiotic resistance due to the presence of resistance-nodulation-division efflux pumps that physically sequester incoming antibiotics^6^. Moreover, biofilms act as protective barrier against antibiotic penetration^7^. Genetically, *P*. *aeruginosa* can also acquire antibiotic resistance through mutations or horizontal transfer of responsible genes^8^.

A variety of antibiotics has been discovered and developed from the 1950s through the 1960s following the commercialization of penicillin^9^. Thereafter, antibiotic-resistant pathogens were increased by indiscriminate use of antibiotics in humans and animals, so development of novel antibiotics for treating AMR infections has been actively pursued, but has not been successful^10,11^. Such present situation calls for efforts on various levels in order to better manage AMR infections.

Previous studies aimed at elucidating antibiotic resistance mechanisms have had the following limitations. First, many studies have been based mainly on comparative genomic analysis to find causes of antibiotic resistance^12,13^. Second, transcriptome- and proteome-level responses have been explored using antibiotic-susceptible laboratory *P*. *aeruginosa* strains^14–17^. Therefore, physiological characteristics of antibiotic-resistant clinical isolates have been less considered. In the present study, we sequenced the whole genomes of three MDR *P*. *aeruginosa* clinical isolates and profiled their genome-wide RNA transcripts as well. Function-level categorization of differentially expressed genes provided initial insights into their virulence potentials, which were validated later experimentally. Further, RNA-level analysis enabled us to understand how MDR strains respond to antibiotic exposure. Results provided in the present study will contribute to expand the current understanding of the physiological characteristics of antibiotic-resistant *P*. *aeruginosa* strains and the mechanisms of antibiotic resistance.

## Results

### Comparative genomic analysis of clinically isolated MDR *P*. *aeruginosa* strains

Bacterial adaptations resulting in antibiotic resistance are caused by DNA-level changes^18^. To elucidate those modifications uniquely observed in MDR strains, we sequenced the whole genomes of three MDR strains (Y71, Y82, and Y89) isolated from independent pneumonia patients. As a negative control, we also sequenced the genome of an antibiotic-susceptible isolate (Y31) recovered from a patient also diagnosed with pneumonia. Minimum inhibitory concentration (MIC) values of five different antibiotics against the strains were determined as shown in Table 1. PAO1, a standard laboratory *P*. *aeruginosa* strain, was used as a control in the MIC test, and its genome was used as a reference in subsequent sequencing analyses. The four clinical isolates originated from different patients and were confirmed to be phylogenetically distant from one another based on the random amplified polymorphic DNA assay^19^.

**Table 1:** Strains used in this study and their resistance against five different antibiotics.

Strain	MIC range (µg/ml)^a^				
	TIC	TOB	IMI	CIP	PIP/TZP
PAO1	32	0.25	4	0.0625	8(4)
Y31	128	0.25	4	0.5	8(4)
Y71	1024	128	16	16	128(4)
Y82	1024	256	16	16	64(4)
Y89	1024	128	16	16	128(4)

^a^Minimum inhibitory concentration (MIC) was determined by the broth dilution method at 37 °C for 24 h. TIC: ticarcillin; TOB: tobramycin; IMI: imipenem; CIP: ciprofloxacin; PIP: piperacillin; TZP: tazobactam.

**Figure 1:**
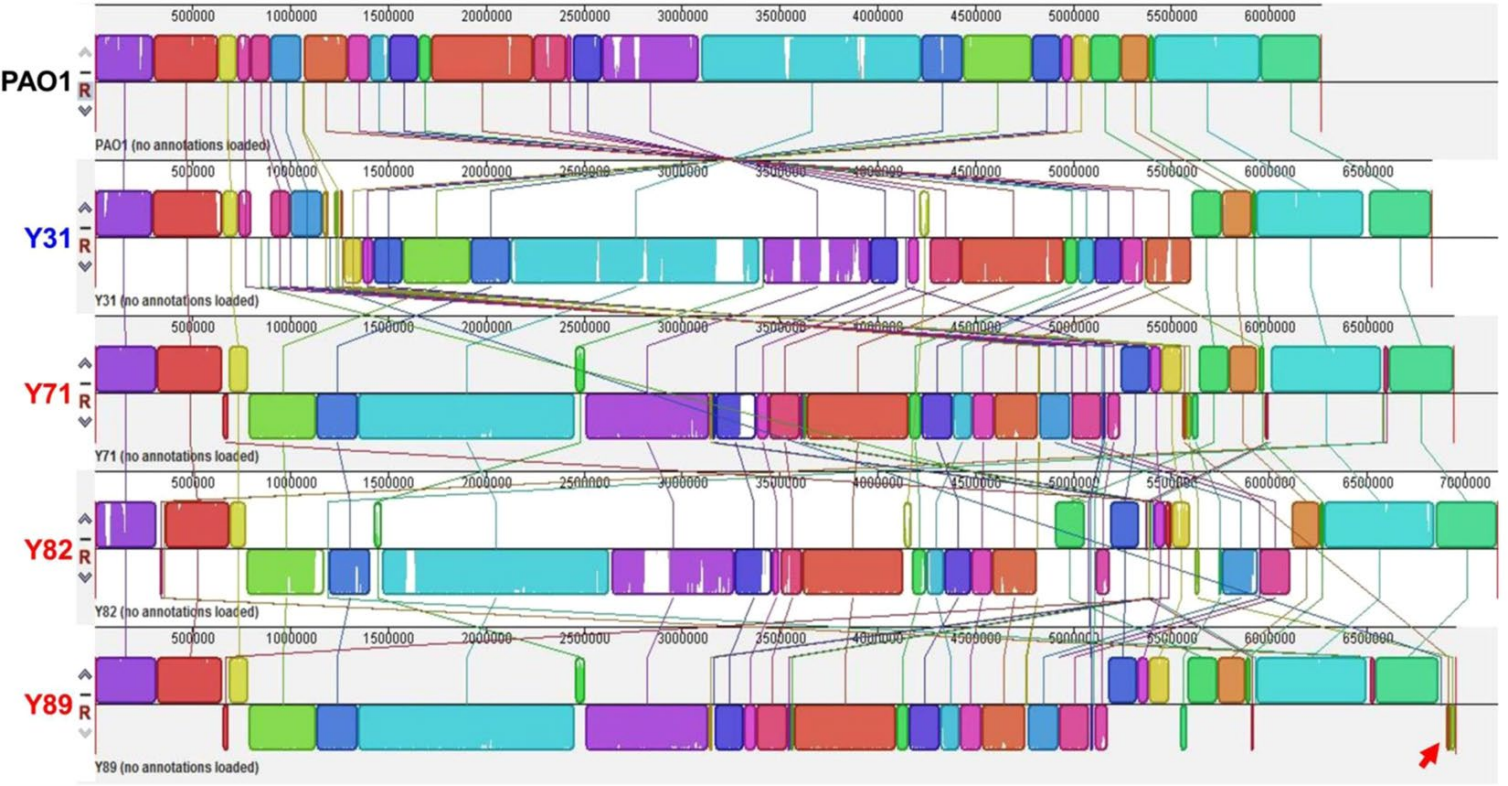
Alignment of PAO1, Y31, Y71, Y82, and Y89 whole genomes with the Mauve program. Strain names are indicated on the left in different colors. The Y89 strain harbors a plasmid, and its sequence is shown at the end (red arrow). The PAO1 genome is displayed as a collection of squared regions above the center line. Regions with homologous sequences are shown with the same-colored squares. Squares that lie above the center line are aligned in the same orientation, while squares below the line are aligned in the inverse orientation, relative to the corresponding regions of the PAO1 genome. Each squared region consists of a collection of vertical lines, and the height of each line is drawn proportional to the similarity between the sequences. Thus, a white region inside squared regions represents a sequence that is exclusively present on a given genome.

Assembled genomes were constructed and aligned with the PAO1 genome (Fig. 1). Inversion of a large region was invariably observed in the middle of every genome. The overall information for all genomes is summarized in Table 2. While the PAO1 genome is about 6.26 Mbp and harbors 5,572 predicted open reading frames (ORFs)^20^, all clinical isolates were found to have larger genomes and more ORFs. The presence of a plasmid was only identified in the Y89 strain, as shown by a red arrow (Fig. 1).

**Table 2:** General genome information for the five strains used in the study.

Strain	Genome Size (bp)	DNA GC (%)	Total genes	Protein-coding genes	Pseudo genes	rRNA genes	tRNA genes	Prophage regions
PAO1	6,264,404	66.60%	5,697	5,572	19	13	63	2
Y31	6,831,076	66.15%	6,313	6,190	42	12	65	6
Y71	6,940,949	65.97%	6,477	6,335	61	12	65	7
Y82	7,106,857	65.75%	6,504	6,383	44	12	63	4
Y89	6,868,832	65.98%	6,462	6,329	56	12	63	5

DNA GC: proportion of DNA that is guanine or cytosine; rRNA: ribosomal RNA; tRNA: transfer RNA.

Next, we sought to examine whether MDR strains possess genes responsible for antibiotic-resistant phenotypes. To this end, we performed blastp analysis using genome-deduced proteins against the ResFinder and Comprehensive Antibiotic Resistance Database (CARD) databases^21,22^. MDR strains, but not antibiotic-susceptible strains, contain well-characterized antibiotic-resistant genes (ARGs). Table 3 shows that the *blaOXA-1* gene, encoding an enzyme for hydrolysis of β-lactam antibiotics^23^, is present in all three MDR strains. The *aadB*^24^, *aac(6′)-31*^25^, and *aph(3′)-VIa*^26^ genes, conferring aminoglycoside resistance, were found in Y71 and Y89, whereas the Y82 strain harbored the *aadB* and *aac(6′)-31* genes. The presence of these additional genes was reflected in their tobramycin resistance, with the MICs of tobramycin being increased more than 512-fold compared to those of PAO1 and Y31 (Table 1). All MDR strains possessed the *sul1* gene encoding a sulphonamide-resistant dihydropteroate synthase^27^. The *cmx* gene, whose product confers chloramphenicol resistance^28^, was identified in the Y71 and Y89 genomes. Protein sequences encoded by these resistant genes are almost identical to those retrieved in the ResFinder and CARD databases (Table 3), further validating that these proteins perform their known functions.

**Table 3:** Presence of genetic markers for antibiotic resistance in each MDR strain.

Strain	Resistance gene^a^	Gene_ID	Identity (%)^b^	Query/HSP^c^	Phenotype
Y71	*aadB*	Y71_4780	100	534/534	Aminoglycoside resistance
	*blaOXA-1*	Y71_4781	100	831/831	β-lactam resistance
	*aac(6′)-31*	Y71_4782	98.65	519/519	Aminoglycoside resistance
	*aph(3′)-VIa*	Y71_4787	98.97	780/780	Aminoglycoside resistance
	*sul1*	Y71_4790	100	852/852	Sulphonamide resistance
	*cmx*	Y71_4792	99.83	1176/1176	Phenicol resistance
Y82	*sul1*	Y82_4660	100	852/852	Sulphonamide resistance
	*aac(6′)-31*	Y82_4665	98.65	519/519	Aminoglycoside resistance
	*blaOXA-1*	Y82_4666	100	831/831	β-lactam resistance
	*aadB*	Y82_4667	100	534/534	Aminoglycoside resistance
Y89	*aadB*	Y89_4691	100	534/534	Aminoglycoside resistance
	*blaOXA-1*	Y89_4692	100	831/831	β-lactam resistance
	*aac(6′)-31*	Y89_4693	98.65	519/519	Aminoglycoside resistance
	*aph(3′)-VIa*	Y89_4698	98.97	780/780	Aminoglycoside resistance
	*sul1*	Y89_4701	100	852/852	Sulfonamide resistance
	*cmx*	Y89_4703	99.83	1176/1176	Phenicol resistance

^a^These antibiotic-resistant genes were taken from the ResFinder database and the Comprehensive Antibiotic Resistance Database (CARD). ^b^Identity is the best matching percentage between the antibiotic-resistant gene presented in ResFinder or CARD and the corresponding gene of the input genome. ^c^Query is the gene length based on the database, and HSP is the gene length of the corresponding gene of the input genome.

In addition, a conserved mutation was observed in the MDR strains that results in amino acid replacement T83I in topoisomerase, the product of the *gyrA* gene (Table 4). This particular mutation has been reported to be responsible for quinolone resistance^29–32^, and our MIC test further confirmed that all three MDR strains were highly resistant to ciprofloxacin treatment (Table 1). Moreover, mutations of the *oprD* gene, which encodes a porin through which imipenem can penetrate^29^, were also identified in the three MDR strains. Premature termination of translation ensued due to the mutations (Table 4). Together, genome-wide examinations revealed a wide range of common mutations that contributed to the antibiotic resistance.

**Table 4:** Presence of mutations for antibiotic resistance in each MDR strain.

Strain	Phenotype	Resistant mechanism	
Y71	Fluoroquinolone resistance	Target mutation	*gyrA* T83I
Y82			*gyrA* T83I
Y89			*gyrA* T83I
Y71	Carbapenem resistance	Deletion on *oprD*	297a.a/443a.a
Y82			338a.a/443a.a
Y89			297a.a/443a.a

### Transcriptomic landscapes of MDR *P*. *aeruginosa* strains

**Figure 2:**
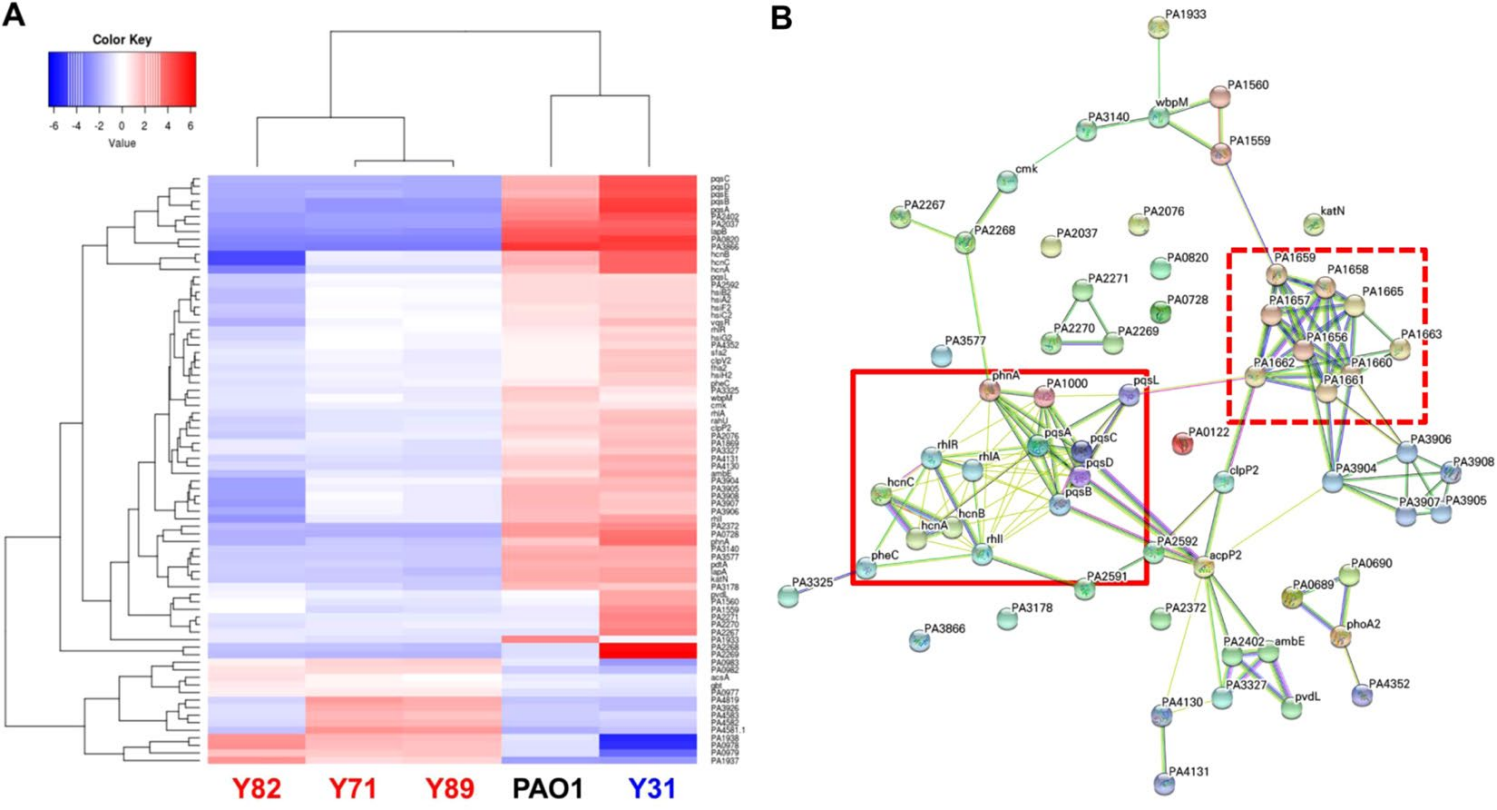
Transcriptome comparison between MDR versus antibiotic-susceptible strains. (**A**) Seventy-eight genes that satisfied two parameters (fold change >2, FDR < 0.05) were selected among the antibiotic-susceptible (PAO1, Y31) and MDR strains (Y71, Y82, Y89) by the edgeR tool. These genes were lined up next to the heat map. TMM-normalized read counts of samples were converted to log2. After each gene was averaged from five samples, the average was subtracted from the read count of each gene. If the last calculated value was high, it was displayed in the heat map in red; when it was low, it was displayed in blue, as shown in the Color Key (top left). Hierarchical clustering was performed on the basis of genes and samples. (**B**) 64 genes that exhibited decreased expression (>2-fold) in the MDR strains were used for a functional gene network using STRING. The box drawn with the solid line was the group related to quorum sensing, and box drawn with the dashed line was the group related to the type VI secretion system.

Previous studies using antibiotic-resistant *P*. *aeruginosa* isolates have focused on identifying DNA-level variations and their effects. To understand better the physiological characteristics of MDR strains, we also conducted RNA sequencing (RNASeq) analysis. Differentially expressed genes with greater than 2-fold increases (or decreases) and a false discovery rate (FDR) less than 0.05 were extracted from both groups (MDR and antibiotic-susceptible [AS]) and used for the construction of a heat map (Fig. 2A). Overall, a larger number of genes exhibited decreased transcriptions in MDR vs. AS strains. Of 78 genes that showed meaningful differences between groups, 64 exhibited decreased expression (more than 2-fold) in the MDR strains. We then constructed a functional gene network using those 64 genes. As shown in Fig. 2B, two distinct clusters stand out in the network. The first cluster (boxed in with a solid line) consists of genes (*rhlI*, *rhlR*, *rhlA*, *pqsA*, *pqsB*, *pqsC*, *pqsD*, *pqsE*, *pqsL*, *phnA*, *hcnA*, *hcnB*, and *hcnC*) related to quorum sensing (QS), a cell density-dependent regulatory mechanism of *P*. *aeruginosa* virulence^33^. The second cluster (boxed in with a dashed line) contains genes encoding components of the type VI secretion system (T6SS) that kills host cells^34^ and competing bacteria^35^. This particular RNASeq analysis was performed using genes that are commonly present in both PAO1 and MDR strains. Therefore, transcriptional responses specific to the MDR strains could not be observed. Nevertheless, our results led us to hypothesize that MDR strains may be less virulent than their AS counterparts.

### RNASeq analysis revealed a unique feature of an MDR strain

**Figure 3:**
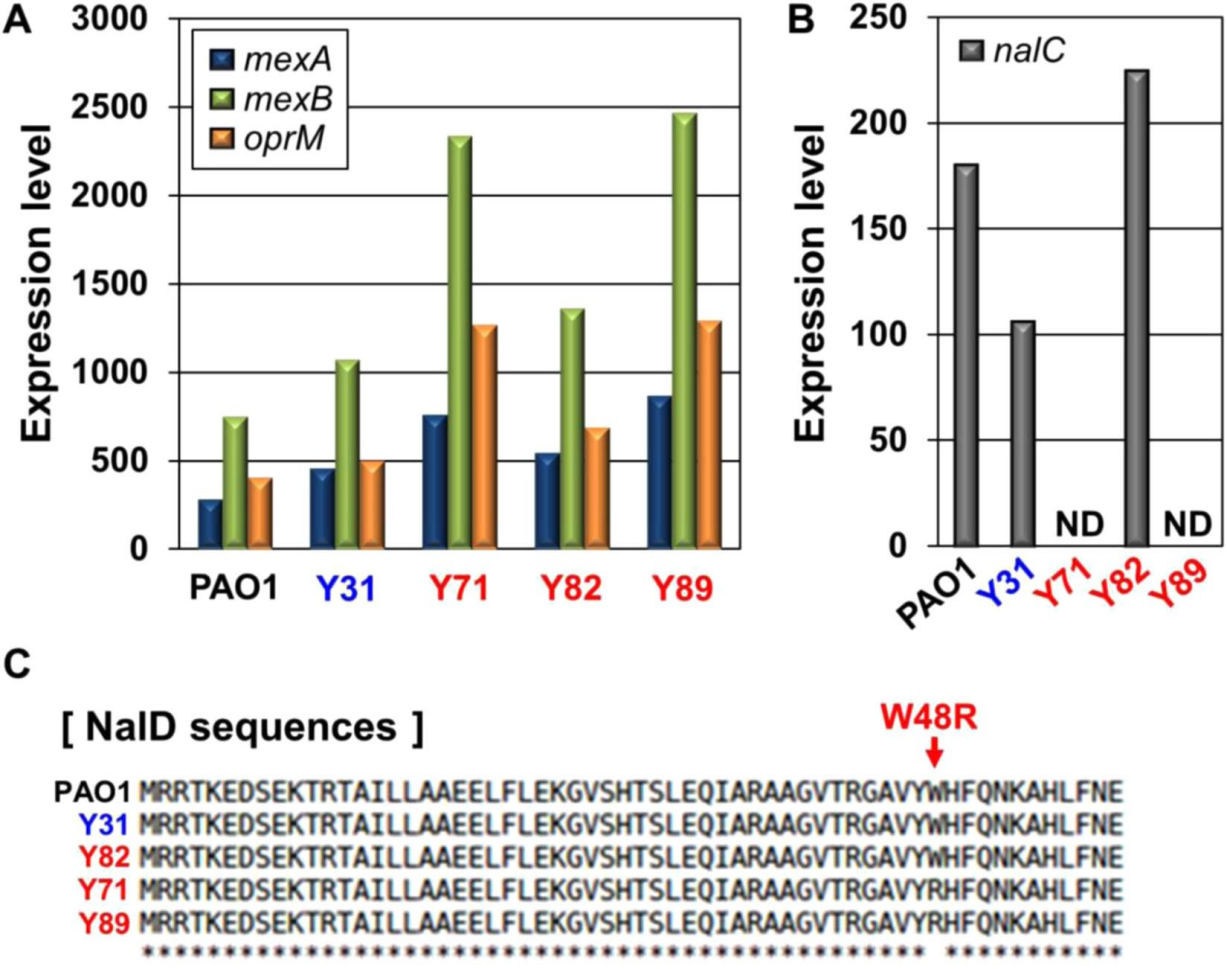
Elevated expressions of *mexAB*-*oprM* genes in Y71 and Y89. TMM-normalized read counts of the *mexAB*-*oprM* operon (**A**) and *nalC* gene (**B**) in RNASeq results with bacteria of the exponential phase. RNA extractions and sequence analysis were performed as described in Experimental Procedures. (**C**) NalD protein sequences of PAO1, Y31, Y71, Y82, and Y89 were compared with CLUSTALW.

The MexAB-OprM efflux pump is the major efflux pump contributing to the non-specific spatial exclusion of incoming antibiotics in *P*. *aeruginosa*. Expression of the *MexAB*-*OprM* operon is controlled by NalC and NalD^36^. Our RNASeq analysis indicated that transcript levels of *mexA*, *mexB*, and *oprM* were not always upregulated in all MDR strains. Among the three MDR strains, Y82 displayed levels of transcription comparable with those of PAO1 and Y31, two antibiotic-sensitive control strains, while the Y71 and Y89 strains exhibited upregulated expression (Fig. 3A). Of note, transcription of the *nalC* gene, encoding a negative regulator of *MexAB*-*OprM* expression, was found to occur actively in Y82 (Fig. 3B). Inactive *nalC* expression was detected in the Y71 and Y89 strains, and these findings are consistent with previous results using other antibiotic-resistant *P*. *aeruginosa* strains^36,37^. In addition, an amino acid substitution mutation (W48R) in NalD protein, another negative regulator of *MexAB*-*OprM* expression^38^, was only detected in the Y71 and Y89 strains (Fig. 3C). These results demonstrate that the Y82 strain has developed antibiotic resistance mechanisms that are independent of the MexAB-OprM system.

### MDR strains were significantly less virulent than the two antibiotic-sensitive strains

#### QS-mediated virulence

**Figure 4:**
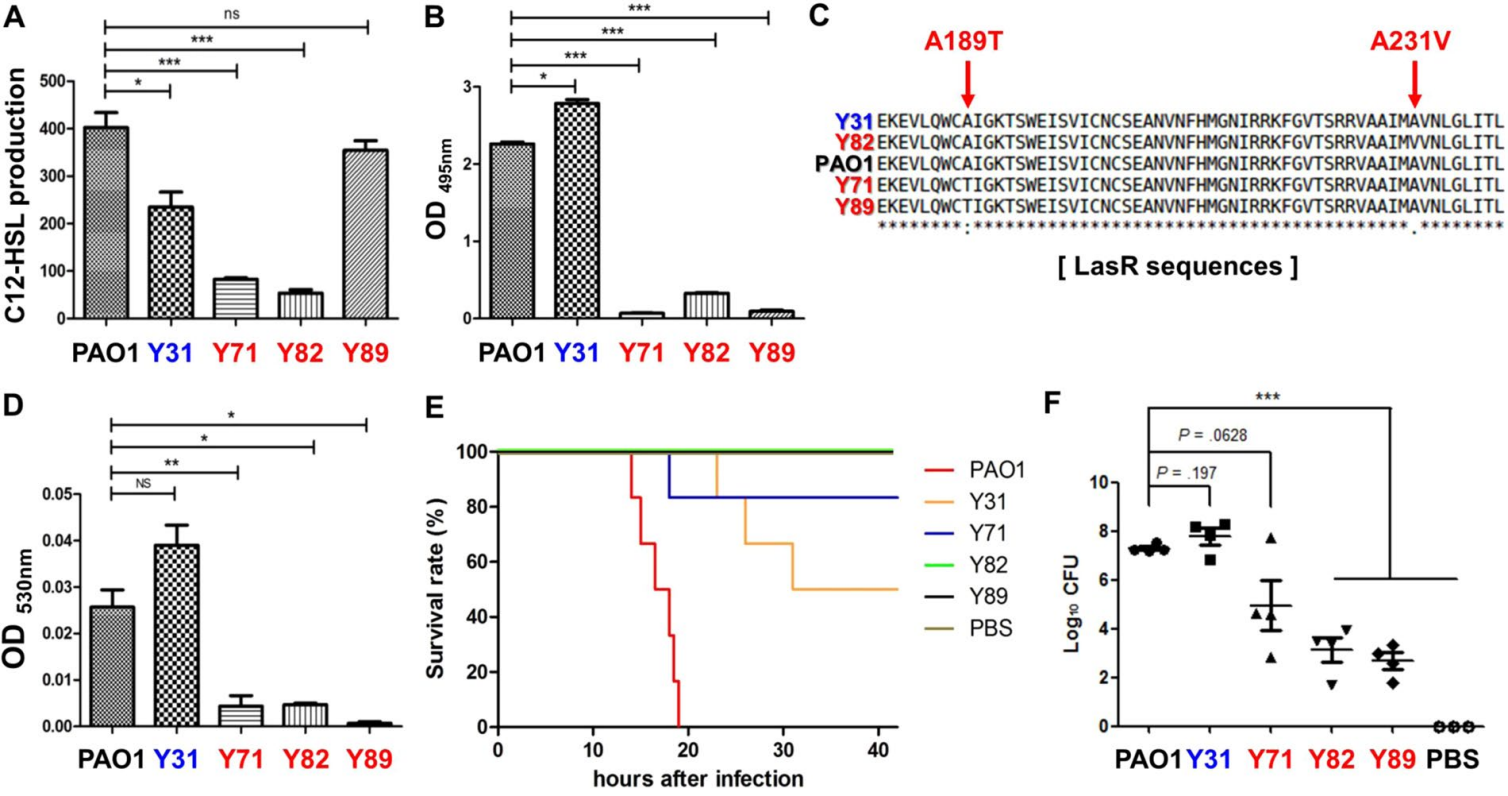
Decreased QS-mediated virulence and *in vivo* infectivity in MDR strains. (**A**) Qualitative analysis of 3-oxo-C12-HSL production. An *E*. *coli* reporter strain harboring the pKDT17 plasmid was incubated with overnight-grown supernatants of each bacterial strain (indicated at the bottom) for one hour and then subjected to β-galactosidase assays. Overnight-grown culture supernatants of each strain were used for elastase (**B**) and pyocyanin (**D**) assays. (**C**) LasR protein sequences of PAO1, Y31, Y71, Y82, and Y89 were compared with CLUSTALW. (**E**) Eight-week-old BALB/C mice (n = 6) were infected with approximately 2.5 × 10^7^ bacterial cells of indicated strains. Mouse survival rates were monitored following infection. (**F**) After the death of mouse or 42 hours of infection, mouse lung was homogenized in PBS and serially diluted for enumerating bacterial cells. Diluted lung homogenates were spotted on Pseudomonas Isolation Agar. ***p < 0.001 vs. the CFU of the PAO1-infected samples.

With the availability of the genome sequences, we compared the promoter sequences of 45 genes selected to be related to QS (Table S1). We also compared the protein sequences of these 45 genes with the corresponding proteins in PAO1. Among the three MDR strains, Y82 was found to exhibit considerable variations in the *lasI*, *phzM*, *hcnABC*, *and aprA* promoter regions, with a complete deletion of the *hcnABC* gene cluster (Table S1), suggesting that mutations in QS-related genes occurred extensively in the Y82 strain. Table S2 shows the transcript levels of the 45 genes in each strain. As was expected from the mutation of the *lasI* gene promoter, Y82 produced significantly lower levels of *lasI* transcript, while active *lasI* gene transcriptions were detected in the two other MDR strains (Table S2, green box). Consistent with this mRNA analysis, Y82 produced the smallest amount of C12-HSL, an autoinducer produced by the LasI protein (Fig. 4A). When culture supernatants were assessed for elastase activity, however, all three MDR strains produced markedly decreased elastase activities (Fig. 4B). This indicates that some other mutations are responsible for the defective elastase production in the Y71 and Y89 strains. Of note, these two MDR strains were found to produce LasR with a point mutation of A189T (Fig. 4C). The 189^th^ and 231^st^ amino acid residues, conserved in LasR, SdiA, and TraR, three different LuxR homologues^39^, fall in the DNA binding domain of the protein^40^. Therefore, the protein with this particular amino acid polymorphism is predicted to be less active in DNA binding. Pyocyanin production, another important event controlled by QS in *P*. *aeruginosa*, was also abrogated specifically in the MDR strains, but not in the PAO1 and Y31 strains (Fig. 4D). Because elastase and pyocyanin are two major QS-mediated virulence determinants, our results strongly suggest that overall QS capability is significantly impaired in MDR strains. Consistent with these *in vitro* results, mice infected with each of the MDR strains exhibited significantly better survival than did the PAO1- or Y31-infected groups (Fig. 4E). Not a single mouse perished by infection with Y82 or Y89, while all six mice were killed by PAO1 at the same infection dose (~2.5 × 10^7^ cells). Moreover, the numbers of bacterial cells recovered from the mouse lungs were markedly decreased in mice infected with Y71, Y82, or Y89 (Fig. 4F), indicating that these MDR strains were cleared by host airway immunity. The mice lung histology results further corroborate that the three MDR strains had attenuated virulence, since the elevated infiltration of immune cells observed in PAO1- and Y31-infected lung tissues indicated that these bacterial strains were more virulent (Fig. S1). These results further confirm that the virulence potentials of our MDR strains were significantly lower than those of the two AS strains.

#### Type VI secretion system (T6SS)

**Figure 5:**
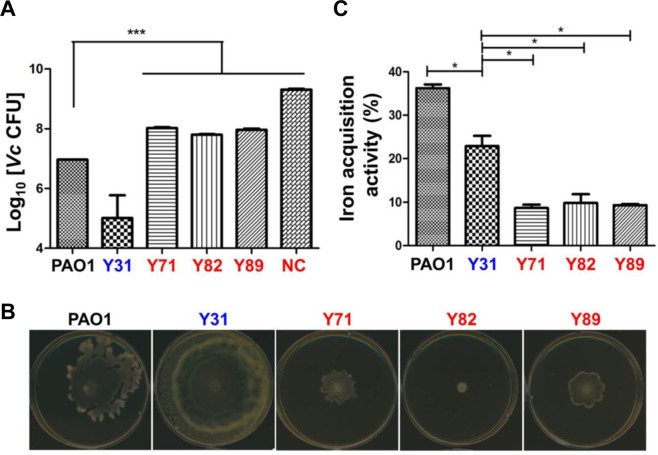
Decreased Type VI secretion system swarming activities and iron acquisition activities in MDR strains. (**A**) Bacterial competition assays with *Vibrio cholerae V52* and *P*. *aeruginosa*. *V*. *cholerae* V52 as prey of *P*. *aeruginosa* strains, PAO1 and clinical isolates were incubated overnight in LB. V52 alone and mixtures of *P*. *aeruginosa* and V52 in a 1:1 ratio were grown on LB agar for 5 hr. Bacteria grown on LB agar plates were scraped, serially diluted, spread on TCBS agar for selection of V52, and incubated overnight at 37 °C for enumeration. NC is CFU of V52 alone for 5 hr. ***p < 0.001 vs. V52 CFUs after incubation with PAO1. (**B**) Overnight-grown *P*. *aeruginosa* strains were spot-inoculated on 0.5% agar plate composed of the 0.8% nutrient broth and 0.5% glucose and incubated overnight at 30 °C. **(C)** CAS assay for iron acquisition activity. The 10-fold diluted supernatants from overnight cultures were reacted with CAS solution for 2 hr, and the OD of the resulting solution was measured at 630 nm. *p < 0.05 vs. iron acquisition activity of PAO1 and Y31.

*P*. *aeruginosa* has three T6SSs composed of H1-, H2-, and H3-T6SS. H1-T6SS plays a role in anti-prokaryotic activity, whereas the H2- and H3-T6SSs target both eukaryotic and prokaryotic cells^41^. The various effector proteins of T6SS pass through Hcp tubes with spikes consisting of VgrG protein at the end and are transferred to target cells^42,43^. Transcriptions of H2-T6SS-related genes (*PA1656*, *PA1657*, *PA1658*, *PA1659*, *PA1660*, *PA1661*, *PA1662*, *PA1663*, and *PA1665*) of the MDR strains were significantly downregulated compared with those of AS strains (Fig. 2). To assess the T6SS-mediated anti-prokaryotic activities of MDR vs. AS strains, we performed a bacterial competition assay using *Vibrio cholerae* V52 strain as prey. The choice of V52 strain, a T6SS-positive strain, was based on a previous report showing that *P*. *aeruginosa* T6SS is induced by T6SS-mediated counterattack^35^. All five *P*. *aeruginosa* strains were capable of killing the prey strain, although the degree of killing varied among the strains (Fig. 5A). Of note, all three MDR strains were less capable of killing the prey strain than were the two AS strains (Fig. 5A). After 5 hr of incubation with each of three MDR strains, ~10^8^ viable *V*. *cholerae* cells were invariably recovered, while ~1.5 × 10^9^ cells were detected when incubated for 5 hr in the absence of a *P*. *aeruginosa* predator strain. In contrast, ~10^7^ and ~1 × 10^5^ prey cells remained viable after the incubations with PAO1 and Y31, respectively (Fig. 5A). It is unclear why the Y31 strain exhibited exceptionally robust killing activity. These results indicate that the predictions proposed from the RNASeq analysis are well validated in our bacterial competition assays.

#### Swarming motility

Bacterial swarming motility is associated with *P*. *aeruginosa* virulence. A mutant defective in swarming motility exhibited attenuated *in vivo* virulence^44^, and virulence-associated genes were actively expressed in swarming bacteria^45^. When bacterial strains were allowed to swarm on 0.5% agar plates, the PAO1 and Y31 strains exhibited robust swarming, while the three MDR strains were less active in their swarming motilities (Fig. 5B).

#### Iron acquisition capability

Iron acquisition is also important for bacterial survival and virulence. *P*. *aeruginosa* produces siderophores for iron uptake, such as pyoverdine and pyochelin, which show high affinities to ferric iron^46^. Figure S2 shows the arrangement of genes involved in the synthesis of pyoverdine and its receptor FpvA in PAO1 and the three MDR strains. Of note, the pyoverdine gene cluster found in PAO1 was not present in the three MDR strains (Table S3). Instead, they commonly possessed a gene cluster, which was predicted to play a similar function (Fig. S2, Table S3). Due to the differences in gene arrangements among strains, it was thought that examining the transcript levels of component genes would not be meaningful. Therefore, we performed a chrome azurol sulphonate (CAS) assay to detect the siderophore activity in bacterial culture supernatants. The three MDR strains exhibited iron acquisition activities substantially lower than those of PAO1 and Y31 (Fig. 5C). Because no major differences in the gene clusters for pyochelin and putative nicotianamine siderophores^46^ were observed between strains (data not shown), the overall reduction in the iron acquisition activities is likely attributed to the defective pyoverdine activities of the MDR strains.

### Transcriptional responses of MDR strains to antibiotic stresses

Our results shown in Table 3 demonstrate the presence of ARGs (antibiotic-resistant genes) in the MDR strains. In addition to these well-characterized ARGs, our comparative genomic analysis also revealed that certain genes were uniquely and commonly present in all three MDR strains. To better understand the roles of these MDR-specific genes in mounting antibiotic resistance, we analyzed how actively and instantly the transcriptions of such genes occur upon antibiotic stresses. To this end, we treated MDR strains with a mixture of three different antibiotics (imipenem, tobramycin, and ciprofloxacin) at sub-MIC concentrations and extracted RNA for RNASeq analysis.

**Figure 6:**
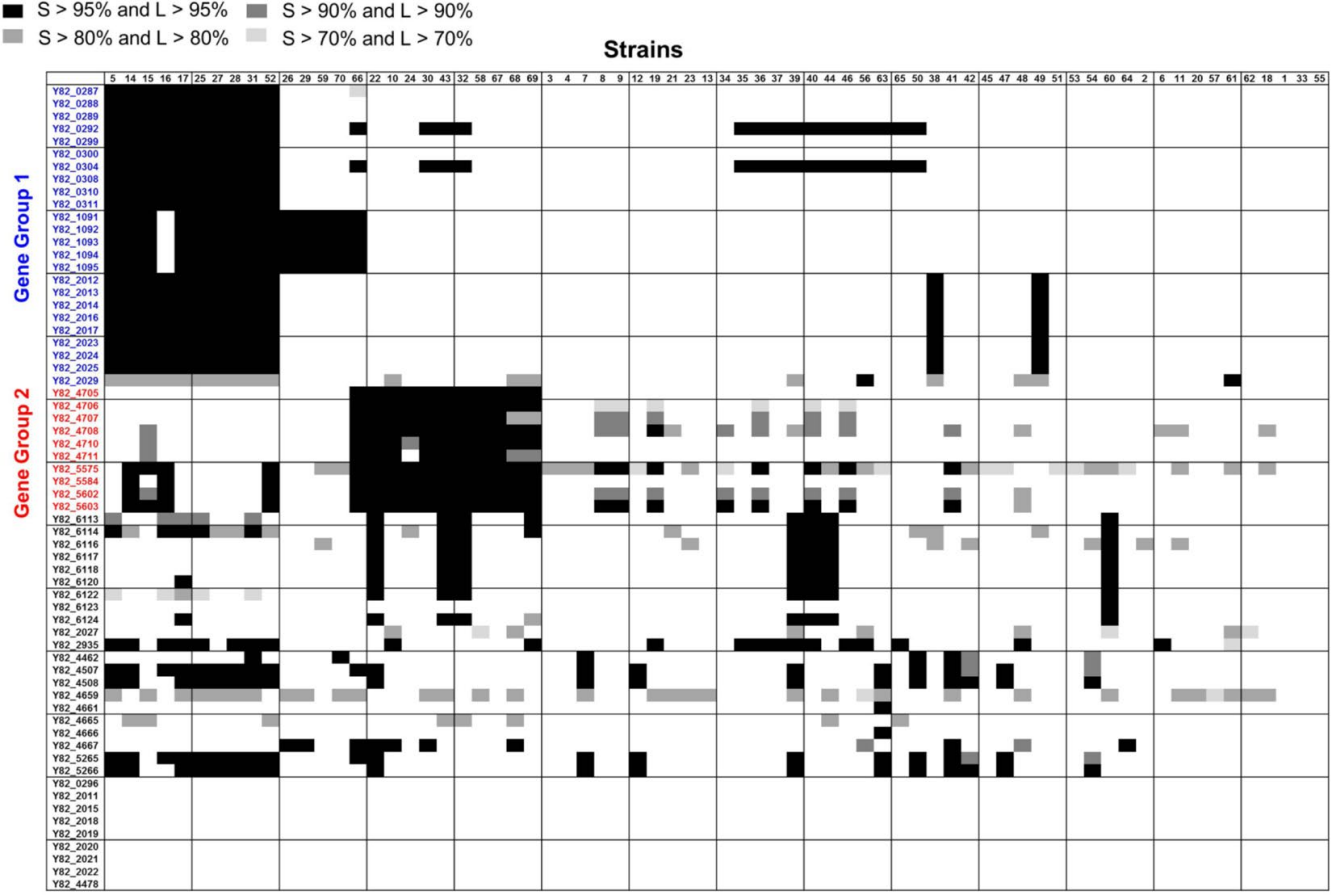
Distribution of 64 ARGs in the genomes of 70 different MDR *P*. *aeruginosa* strains. Protein sequences encoded by the 64 ARGs (far left column) were aligned against all protein sequences deduced from genomes of 70 different MDR *P*. *aeruginosa* strains (top line), with several thresholds of similarity (S) and length coverage (L); (S > 95% and L > 95%, S > 90% and L > 90%, S > 80% and L > 80%, S > 70% and L > 70%). Seventy MDR strains are listed in Table S4 with detailed information. Presence of 64 genes in each genome is indicated with color-coded square boxes. The heat map was constructed by applying the color that satisfied the highest threshold. The blue-colored and red-colored genes were designated as gene group 1 (GG1) and gene group 2 (GG2), respectively.

For this purpose, we sorted out a list of MDR-unique genes through several steps (Fig. S3). First, 169 genes were selected that (i) were invariably present in all three MDR strains and (ii) exhibited active transcriptions as measured by RNASeq analysis. Among the 169 genes, 53 were excluded because they were found to be present in other AS *P*. *aeruginosa* strains, such as PA14^47^ and VRFPA07^48^. Exclusion of these genes was based on clustering under both conditions (>95% sequence similarity with >95% gene length coverage (S95L95) and >50% sequence similarity with >50% gene length coverage (S50L50)) by the Blast-2.2.26 algorithm^49^. Genes that cluster only among MDR strains under both conditions were selected as MDR strain-specific genes. Then, 52 additional genes that are present in prophage clusters and encode transposases were removed from the list, because they were not presumed to be related to antibiotic resistance. Through this process, we selected 64 genes that are specifically present in our MDR strains and predicted to be involved in antibiotic resistance (Fig. S3). Of note is that 55 of these 64 genes were also found to be present in other antibiotic-resistant *P*. *aeruginosa* strains (n = 70) reported elsewhere (Table S4)^12,13,48,50–53^. When the 64 genes were examined for presence in the genomes of these 70 strains, 55 were identified to be present in more than one strain (Fig. 6). At high threshold conditions (>95% sequence similarity with >95% gene length coverage), Y82_0292 and Y82_0304, encoding excinuclease ABC subunit A and glyoxalase (Table S5), were invariably found in the same 25 strains among the 70 reference strains. These two are the most abundantly identified genes. When the threshold was lowered to >70% sequence similarity and >70% gene length coverage, the Y82_5575 gene was found in 44 strains. Y82_4666, which codes for OXA-1 family class D β-lactamase, on the other hand, was present only in strain 63, *P*. *aeruginosa* AR_0108 (Fig. 6). It is of particular interest that 10 genomes of strains 5, 14, 15, 16, 17, 25, 27, 28, 31, and 52 contain a common group of genes (gene group 1, blue font), while another distinct group of genomes (strains 10, 22, 24, 30, 32, 43, 58, 66, 67, 68, and 69) harbors a different subset of genes (gene group 2, red font) (Fig. 6). None of the strains, however, possess these two gene groups together. This result strongly suggests that our MDR strains (Y71, Y82, and Y89) are unique in genome repertoire among antibiotic-resistant *P*. *aeruginosa* strains.

**Figure 7:**
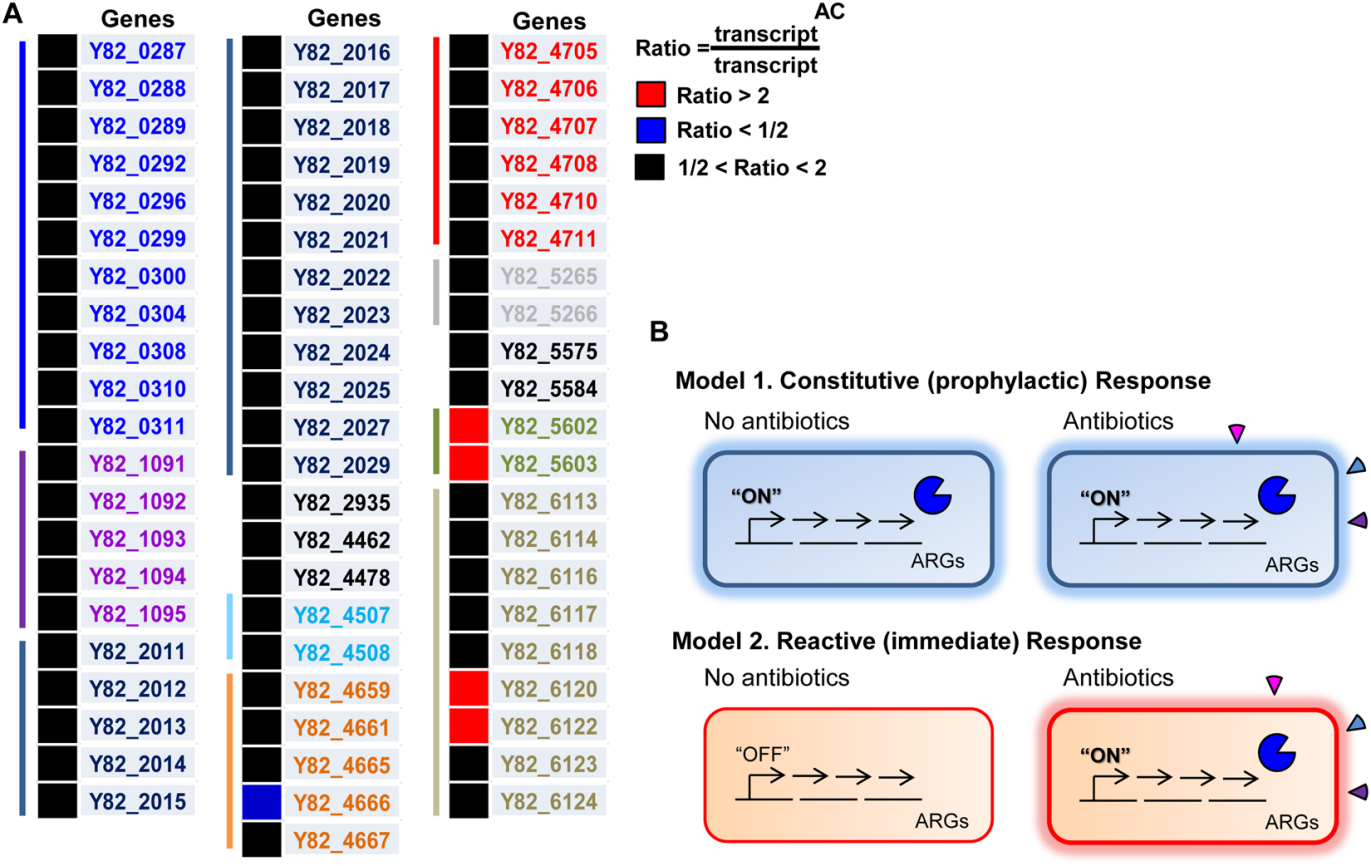
Transcriptional responses of 64 ARGs to the treatment with antibiotic cocktail (AC) and constitutive function of the ARGs to endure antibiotic stresses. (**A**) The mRNA transcript of each of the 64 genes was calculated before and after AC treatment in each MDR strain and the ratio of their average values is represented as a color-coded square. Red and blue squares indicate >2-fold increase or >2-fold decrease in expression upon AC treatment, respectively. The genes with fold changes between 0.5 and 2 are shown in black squares. The names of the representative genes were taken from the strain Y82 that has the largest genome among the MDR strains. Among the MDR strains (Y71, Y82, and Y89), 64 ARGs presented homology of more than 90%. (**B**) Potential working models by which ARGs respond to antibiotic stresses. Pacman shapes represent the expressions of ARGs, and fan shapes represent various antibiotics. The thick cell wall means that the ARG-expressing bacterial cell is ready to withstand antibiotic stresses.

We next calculated the ratios of transcriptions of these 64 genes after vs. before antibiotic cocktail treatment and presented the ratios with color-coded squares (Fig. 7A). Among 64 genes, those encoding *aac(6′)-31* (Y82_4665), *OXA-1* β-lactamase (Y82_4666), and *aadB* (Y82_4667), which are well-characterized ARGs, were ranked highest based on mRNA reads even without antibiotic cocktail (AC) treatment (yellow box, Table S5). Following AC treatment, expression of these three genes was slightly decreased (green box, Table S5). Reflecting these results, color-coded squares are shown as black for Y82_4665 and Y82_4667 and blue for Y82_4666 (Fig. 7A). Similar to these genes, no significant increases in AC-induced transcription were detected in the majority of the 64 genes (Fig. 7A). Only four genes, Y82_5602, Y82_5603, Y82_6120, and Y82_6122, showed elevated transcriptions following AC treatment. Together, these results suggest that transcription of MDR-specific genes does not increase immediately when antibiotic stresses are encountered. Instead, ARGs are constitutively transcribed, albeit in varying degrees, even in the absence of antibiotic stress, rendering MDR *P*. *aeruginosa* strains resistant to future antibiotic attacks (Fig. 7B).

## Discussion

A hallmark of *P*. *aeruginosa* infections is the emergence of antibiotic-resistant variants. Healthcare authorities in many countries have already classified *P*. *aeruginosa* as a dangerous pathogen, especially for patients in critical care, and make strenuous efforts to monitor its infections carefully. Here, we provide insights into the physiological features of MDR *P*. *aeruginosa* clinical isolates and the mechanisms by which they regulate the expression of ARGs. To date, studies to elucidate antibiotic resistance mechanisms have been performed primarily at the genomic level. Our findings, whose initial clues became available by delineating MDR-specific transcriptomic landscapes, add another layer of understanding as to how MDR *P*. *aeruginosa* strains regulate their virulence and respond to antibiotic stresses.

Previous studies have reported conflicting results regarding the relationship between antibiotic resistance and virulence of *P*. *aeruginosa* clinical isolates. A group of antibiotic-resistant *P*. *aeruginosa* isolates exhibited reduced QS-related virulence and attenuated capabilities to disrupt intestinal epithelial cell barrier^54,55^. Similar to our results, Gómez-Zorrilla and colleagues showed that mice infected with drug-resistant strains survived better than those infected with antibiotic-sensitive control strains^56^. This trend was evident not only in the analysis of naturally isolated strains, but was also observed with artificially produced mutant strains. A *P*. *aeruginosa* mutant of the *mexR* gene that overexpressed the MexAB-OprM efflux pump produced lower amounts of phenazines and proteases^57^. Moreover, an *oprD* deletion mutant with a phenotype of decreased carbapenem uptake displayed lower virulence *in vivo* than the wild type PAO1^58^, but not in PA14 background^59^. Likewise, antibiotic-resistant variants of *Acinetobacter baumannii*, another Gram-negative bacterial species of clinical importance, harbor a plasmid containing ARGs and a gene encoding a negative regulator of T6SS as well^60^. On the other hand, there are a number of studies claiming that antibiotic-resistant *P*. *aeruginosa* strains are more virulent. ExoU, an effector delivered by a type III secretion system, is a major virulence determinant that causes alveolar epithelial injury^61^. A molecular epidemiological study demonstrated that the *exoU* gene is present more frequently in MDR isolates than in non-MDR isolates^62,63^. Agnello and colleagues also revealed that a positive correlation exists between the prevalence of the *exoU* gene and fluoroquinolone resistance^64^. Patients infected with *exoU*-positive MDR isolates displayed very severe clinical symptoms including lower healing rates^65^. Moreover, a higher cytotoxicity toward cultured epithelial cells was observed in exoU-positive MDR isolates than exoS-positive MDR isolates^55^. Taken together, these results suggest that the *exoU* gene could be an important marker for predicting the virulence of MDR *P*. *aeruginosa* strains. The MDR strains explored in the present study, however, do not possess the *exoU* gene (data not shown). Bacterial evolution occurs in the presence of various selective pressures and proceeds in a direction to increase survival fitness. In this regard, it will be important to ask how DNA mutations resulting in antibiotic resistance affect bacterial virulence, and vice versa.

Our sequence analyses at genome and transcriptome levels were complimentary to each other and helped us to identify a novel mutation leading to antibiotic resistance. A MexAB-OprM efflux pump that spatially excludes incoming antibiotics plays an important role in conferring antibiotic resistance in *P*. *aeruginosa*^66^. In Y71 and Y89, the transcript levels of genes coding for the MexAB-OprM system were increased, as measured by our RNASeq analysis (Fig. 3). The MexAB-OprM operon is negatively regulated by global regulator *nalC*, which also negatively regulates expression of the *PA3719* gene. If the *nalC* gene is deleted, expression of PA3719 increases, and this product binds to MexR, resulting in inhibition of MexR function as a suppressor of *MexAB*-*OprM* expression^67^. In Y71 and Y89, however, despite the *nalC* gene deletion, PA3719 gene transcripts are similar or slightly less than those in PAO1 and Y31 (data not shown). Thus, the increased expression levels of *MexAB-OprM* genes in Y71 and Y89 were not attributed to the elevated production of PA3719 resulting from the deletion of *nalC*. This eventually led us to examine the DNA sequences and mRNA levels of the *nalD* gene (PA3574) encoding another repressor of the MexAB-OprM system^38,68^. In these two strains, the *nalD* gene was found to have a previously undescribed mutation causing a single amino acid substitution (W48R) (Fig. 3C). Because the 48^th^ tryptophan residue is a conserved amino acid in a putative DNA-binding motif of the protein^38^, the mutant protein may be incapable of binding to the *MexAB*-*OprM* promoter. Through the process described so far, we successfully identified a novel mutation associated with the acquisition of antibiotic resistance, and this was made possible because both genomes and transcriptomes were analyzed together.

We postulated that antibiotic resistance in MDR strains would be activated via two different routes, which we termed “reactive” vs. “constitutive” responses, depending on the temporal regulation of ARG expression. In the reactive response model, expression of ARGs was activated when antibiotic stresses were imposed, whereas ARGs were prophylactically expressed in the constitutive response model (Fig. 7B). The reactive response model was proposed based on an idea that activating antibiotic resistance mechanisms is likely an energy-intensive process. Our results strongly support that antibiotic resistance is mediated mainly by a constitutive response for the following reasons. First, the 64 genes were actively transcribed even when grown in plain Luria-Bertani (LB) medium without antibiotics. Second, no additional increases in transcription took place in 60 genes except for that of four genetic elements (Y82_5602/Y82_5603 operon, Y82_6120, and Y82_6122). Long-term exposure to high concentrations of antibiotics results in acquisition of antibiotic resistance. During this process, ARGs must have evolved in such a way that their expression was controlled by promoters with constitutive activity. With regard to this, it will be important to address (i) how constant transcription of ARGs affects bacterial fitness under normal conditions and (ii) whether reduced virulence detected in MDR strains is associated with persistent expressions of ARGs.

In this study, we expanded our understanding of the 64 genes considered to be involved in antibiotic resistance. Of these 64 genes, 55 were found in 67 of 70 antibiotic-resistant *P*. *aeruginosa* strains reported in other studies (Fig. 6). These results demonstrate that the 64 genes that we sorted may indeed be closely related with antibiotic resistance in *P*. *aeruginosa*. Through the genome-wide comparisons against the genomes of 70 antibiotic-resistant clinical isolates, we identified two major gene groups (GG1 and GG2) that are prevalently present in the genomes of antibiotic-resistant *P*. *aeruginosa* isolates. Interestingly, most of the 70 reference strains carry either GG1 or GG2, but not both (GGs), while the two GGs co-exist in our MDR strains (Y71, Y82, and Y89). Although it is not possible to compare antibiotic resistance phenotypes directly between these 70 strains and our MDR strains, the 70 strains were determined to be resistant to three different classes of antibiotics^12,13,48,50–53^. On the other hand, the MDR strains explored in the current study are resistant to at least five different antibiotics (Table 1). Therefore, one important question is whether stronger resistance to wider ranges of antibiotics is achieved in the presence of both GGs. Addressing this question may provide a novel genetic trait that could potentially be used as a marker to predict the resistance of a *P*. *aeruginosa* clinical isolate.

In conclusion, we extended the understanding of the physiological characteristics of MDR *P*. *aeruginosa*. The MDR strains analyzed in this study exhibit not only a conserved set of genetic variations responsible for antibiotic resistance, but also a distinct pattern of ARG distribution, which can potentially be used as a marker for MDR prediction. Furthermore, RNASeq analysis provided novel insights into their virulence characteristics and transcriptional responses to antibiotic stresses. We hope that the results provided in the present study will stimulate future investigations to devise new strategies to diagnose and treat MDR *P*. *aeruginosa* infections.

## Experimental Procedures

### Experimental ethics

Experiments using human subjects and experimental animals were performed in strict accordance with guidelines provided by Yonsei University. Protocols were reviewed and approved by Institutional Review Board of Yonsei University College of Medicine. The permit number for mouse infection experiment is 2011-0173-2.

### Bacterial strains and growth conditions

Four clinical isolates (Y31, Y71, Y82, and Y89) were derived from the sputa of four different pneumonia patients, and PAO1 was used as a reference strain. Bacterial cultures were grown in Mueller Hinton (MH) broth (Difco) and LB medium (1% [w/v] tryptone, 0.5% [w/v] yeast extract, and 1% [w/v] sodium chloride) at 37 °C. All bacterial single colonies on LB or MH plates were picked and inoculated in LB or MH broth for precultures and grown overnight. Precultures were diluted 100-fold in fresh LB or MH broth for subculture and incubated at 37 °C and 230 rpm. The incubation time was dependent on the experimental procedures.

### Antibiotic susceptibility test (MIC test)

The antibiotic susceptibility test was performed by the broth dilution method^69^, and ticarcillin (Sigma), tobramycin (Sigma), imipenem (Sigma), ciprofloxacin (Fluka), piperacillin (Sigma), and tazobactam (Sigma) for adjuvant of peperacillin were used. PAO1 and the four isolates were precultured and subcultured in MH broth depending on the bacterial strains and growth conditions presented above. Subcultures were incubated at 37 °C and 220 rpm for 3 hr to reach the exponential phase. The bacterial medium was adjusted to optical density (OD) 0.1 (the OD 0.1 of all bacteria is approximately 1 × 10^8^ colony-forming units [CFU]) using fresh MH broth, and this medium was diluted 100-fold and adjusted to a final density of 1 × 10^6^ CFU. All antibiotics were diluted in MH broth to make 2 × concentrations. The final bacterial medium (1 ml) and antibiotic-dissolved media (1 ml) were mixed, and these mixtures were incubated overnight at 37 °C and 230 rpm. The MIC results were determined using the Clinical and Laboratory Standards Institute (CLSI) guidelines.

### Genome sequencing and analysis of four isolates

Clinical isolates were precultured and subcultured in LB medium depending on the bacterial strains and growth conditions presented, and these subcultures were incubated for 3 hr. To extract high quality bacterial DNA, a genomic DNA extraction kit (Intron) was used following the manufacturer’s protocol. Whole genome sequencing of isolates was performed using PacBio RS ll single-molecule real-time (SMRT) sequencing technology. De novo assembly was conducted using the hierarchical genome-assembly process pipeline of the SMRT Analysis v2.3.0. Protein-coding genes were predicted by Prodigal v.2.6.1, and BLAST searches were performed against the UniProt, Pfam, and COG databases^70–72^ to annotate predicted genes functionally. Ribosomal RNA and transfer RNA were predicted using Rfam v12.0^73^. Prophage regions were identified using the PHAST web-based program^74^. Whole genome alignment of PAO1 and the four isolates was performed using Mauve^75^. ARGs were found by blastp with the ResFinder database^21^ and Comprehensive Antibiotic Resistance Database(CARD)^22^. Identity means the best matching percentage between an antibiotic resistance gene and the corresponding sequence of input genome. Query is the length of the antibiotic resistance gene in the database, and HSP is the length of the corresponding sequence of input genome. Sequences of *gyrA*, *oprD*, *nalD*, and *lasR* were analyzed by CLUSTALW^76^. The similarities of promoter regions and ORFs of QS-related genes were compared with PAO1 on nucleotide and amino acid levels.

### RNASeq analysis

PAO1 and clinical isolates were precultured and subcultured in LB medium depending on the bacterial strains and growth conditions presented. Subcultures were grown to the exponential phase, diluted to a final OD of 0.07, and incubated for 1 hr. In the antibiotic-treated group, an AC composed of imipenem, tobramycin, and ciprofloxacin was administered at a final OD of 0.07 for Y71, Y82, and Y89 for 1 hr. A combination of imipenem (1 μg/ml), tobramycin (16 μg/ml), and ciprofloxacin (0.5 μg/ml) was used for stimulation on Y71 and Y89, but a 2-fold lower AC was used with Y82. Aliquots of each culture (n = 3) were pooled together in single tubes for RNA extraction. To extract high quality bacterial RNA, an RNeasy Protect kit (Qiagen) was used with an RNeasy Mini kit (Qiagen) following the manufacturer’s protocol. The isolated RNA was stored at −80°C until use. The Ribo-Zero rRNA removal kit (Epicentre, USA) was used for ribosomal RNA depletion according to manufacturer instructions. Libraries for Illumina sequencing were made with the TruSeq Stranded mRNA sample prep kit (Illumina, USA) following the manufacturer’s protocol. RNA sequencing was performed on the Illumina HiSeq2500 platform using single-end 50 bp sequencing (Chunlab, Seoul, Korea). The raw data from RNA sequencing was aligned to the PAO1 (National Center for Biotechnology [NCBI] Information Bio-Project accession number PRJNA331) and Y82 whole genome sequence by Bowtie 2 version 2.2.6^77^. Differential expression analysis was performed using edgeR, and normalization between the samples was accomplished with the trimmed mean of M values (TMM) method^78,79^. Genes with FDR <0.05 and fold change >2 were identified as differentially expressed and these genes were converted to log2. After subtracting the average expression levels from those of each strain, clustering was performed, and heat maps were created. Network analyses were performed with 2-fold low expressed genes in the MDR strains compared to PAO1 and Y31 using STRING^80^.

### Elastase, pyocyanin, and swarming assays

Production of elastase and pyocyanin and swarming assays were performed following procedures described elsewhere^81–83^.

### Bacterial competition assay

PAO1, clinical isolates, and *Vibrio cholerae* V52 to be used as prey were incubated overnight in LB medium. The bacterial cultures were washed with fresh LB medium and adjusted to 1 ml of OD 1. The bacterial supernatant was removed by spin down and suspended with 100 μl of fresh LB medium. As negative control, 10 μl of V52 were inoculated individually on LB agar plates. As competitive groups, each strain of *P*. *aeruginosa* was mixed 1:1 with V52 (10 μl + 10 μl), and then 20 μl were inoculated on LB agar plates, and all plates containing only V52 were incubated at 37 °C for 5 hr. Bacteria grown on LB agar plates were scraped with a sterile scraper and serially diluted with PBS. The serially diluted media of V52 alone and the co-cultured bacteria were spread on thiosulfate citrate bile salts sucrose (TCBS) agar, a medium for selective growth of *V*. *cholerae*, and incubated overnight at 37 °C.

### Chrome azurol sulphonate assay

The CAS assay was performed in the same way as in a previous study^84^. PAO1 and clinical isolates were precultured and subcultured in LB medium depending on the bacterial strains and growth conditions presented. Subcultures were grown overnight and spun down to obtain supernatant. The overnight supernatant was diluted 10-fold and reacted with CAS solution for 2 hr. The OD of this resultant solution was measured at 630 nm.

### Quantification of 3-oxo-C12-HSL (PAI-1)

To monitor the 3-oxo-C12-HSL, *Escherichia coli* DH5α harboring a reporter plasmid pKDT17^85^ was used. The PAO1 and clinical isolates were precultured and subcultured in LB medium depending on the bacterial strains and growth conditions presented. Subcultures were grown overnight. The supernatant was obtained and filtered with a syringe filter with a 0.2 μm-pore hole. *E*. *coli* pKDT17 was grown overnight in LB medium containing 100 μg/ml ampicillin (Sigma) at 37 °C, washed with fresh LB medium, and adjusted to an OD at 600 nm (OD_600_) of 0.15. One milliliter of supernatant of *E*. *coli* pKDT17 of OD_600_ 0.15 was removed, and *E*. *coli* pellets were suspended in each supernatant of *P*. *aeruginosa* and incubated at 37 °C for 1 hr. Cultured *E*. *coli* was used for β-galactosidase assays to detect an exogenous source of 3-oxo-C12-HSL^85^.

### Sorting antibiotic-resistant genes (ARGs) and transcriptional analysis

In total, 169 genes that satisfied both conditions related to expression level (PAO1 < 3, Y31 < 3, Y71 >10, Y82 >10, Y89 >10) were selected by RNASeq results aligned with the Y82 whole genome. All protein sequences of PA14 and VRFPA07 that were known antibiotic susceptible strains (NCBI Bio-Project accession numbers: PRJNA386 and PRJNA230365) were downloaded from the pseudomonas.com website^86^. The 169 genes and all protein genes of PA14 and VRFPA07 were clustered in both conditions of similarity 95% with length 95% (S95L95) and similarity 50% with length 50% (S50L50) by blastclust^49^. Genes that clustered only among the MDR strains under both conditions were selected as MDR-specific genes (116 genes). The prophage regions found using the PHAST website and genes annotated with transposase that seemed unrelated to antibiotic resistance were excluded, and finally, 64 ARGs that were possibly related with antibiotic resistance were selected. The fold changes of 64 genes between the AC- treated and untreated groups were calculated by dividing their expression levels by the average expression level of each gene.

### Distribution of 64 ARGs in 70 antibiotic-resistant *P*. *aeruginosa* clinical isolates

The 70 genome sequences of MDR *P*. *aeruginosa* clinical isolates were selected from the PATRIC website (64 strains)^50^ and individual studies (6 strains)^12,13,48,51–53^. The 64 ARGs and all protein sequences of the 70 MDR isolates were clustered in four conditions by blastclust (similarity 95% and length 95%, similarity 90% and length 90%, similarity 80% and length 80%, similarity 70% and length 70%). Each threshold was coded with a different color, and a heat map was made by covering the highest color clustered with ARG.

### *In vivo* mouse airway infection

Eight-week-old male BALB/C mice were infected with the bacteria. Each strain was precultured in LB medium overnight, diluted 100 times with fresh LB medium, and incubated to the exponential phase state. Bacterial cells were washed with PBS, and mice were infected with 2.5 × 10^7^ CFU bacteria by intranasal inoculation after being anesthetized intraperitoneally with a mixture of zoletil and rompun. Six mice per group were infected with the bacteria, and five mice were used for the PBS (negative control) group. Mouse lung was removed after the animal’s death or 42 hours of infection. These lungs were fixed in 10% formalin and stained with hematoxylin and eosin. To confirm the colonized bacteria in the mouse lungs, each removed lung was soaked in PBS and homogenized. These homogenized lungs were serially diluted and spotted on Pseudomonas Isolation Agar plates to confirm CFU.

### Statistical Analysis

The data are expressed as the means ± standard deviations. Unpaired Student’s t-tests (two-tailed, unequal variance) were used to analyze the differences between experimental groups. P-values less than 0.05 were considered statistically significant. All experiments were repeated for reproducibility.

## Supplementary information

Supplementary information

## Data Availability

The complete genome sequences of Y31, Y71, Y82, Y89, plasmid of Y89 have been deposited in the NCBI with accession number CP030910, CP030911, CP030912, CP030913 and CP030914. The transcriptome data have been deposited in the bioproject via PRJNA479711.
