# Supplementary material for: Virulence Characteristics and an Action Mode of Antibiotic Resistance in Multidrug-Resistant Pseudomonas aeruginosa
Source: Sci Rep. 2019 Jan 24;9:487. doi: 10.1038/s41598-018-37422-9 (PMC6345838; doi:10.1038/s41598-018-37422-9)
Supplement: Supplementary file 1 — Supplementary information [file 41598_2018_37422_MOESM1_ESM.pdf]

Supplementary Materials for

Virulence Characteristics and an Action Mode of Antibiotic Resistance in Multi-drug Resistant *Pseudomonas aeruginosa*

Wontae Hwang[1] and Sang Sun Yoon[1,2*]

This PDF file includes:

Tables S1 to S5
Figures S1 to S3

PA number	gene	Sequence similarity of QS-related genes							
		Y31		Y71		Y82		Y89	
		promoter	ORF	promoter	ORF	promoter	ORF	promoter	ORF
PA1430	*lasR*	0.997	1	0.9966	0.997	0.9933	0.997	0.9933	0.997
PA1432	*lasI*	0.988	1	0.9878	1	0.17	1	1	1
PA3476	*rhlI*	0.944	0.993	0.9441	0.993	0.9441	0.993	0.9385	0.993
PA3477	*rhlR*	0.992	1	0.9919	1	0.9919	1	0.9919	1
PA0996	*PqsA*	0.997	0.997	0.9966	1	0.9966	1	0.9966	1
PA0997	*PqsB*		0.999		1		1		1
PA0998	*PqsC*		1		0.998		1		0.998
PA0999	*PqsD*		0.995		1		0.998		1
PA1000	*PqsE*		1		1		1		1
PA1001	*PhnA*	0.991	0.989	0.9914	0.993	0.9829	0.996	0.9829	0.993
PA1002	*PhnB*		1		1		1		1
PA1003	*MvfR*	0.993	1	0.9966	0.998	0.9966	1	0.9966	0.998
PA2587	*PqsH*	0.993	1	0.9866	0.995	0.9933	1	0.9866	0.995
PA2302	*AmbE*	0.989	0.99	0.9894	0.993	0.9894	0.994	0.9894	0.993
PA2303	*AmbD*		1		0.998		0.997		0.998
PA2304	*AmbC*		0.996		0.994		0.996		0.994
PA2305	*AmbB*		0.996		0.996		0.996		0.996
PA2306	*AmbA*		1		0.997		0.997		0.997
PA1871	*lasA*	0.996	0.99	0.9964	0.99	0.9964	0.995	0.9964	0.99
PA3724	*lasB*	0.997	0.998	0.965	0.996	0.965	0.998	0.9615	0.996
PA3478	*rhlB*	0.993	1	0.9866	0.998	0.9866	0.998	0.9866	0.998
PA3479	*rhlA*		0.996		0.996		0.996		0.996
PA0051	*phzH*	0.963	0.998	0.9466	0.998	0.9533	0.998	95	0.998
PA1899	*phzA2*	0.987	0.997	0.9833	0.997	0.9833	0.997	0.98	0.997
PA1900	*phzB2*		1		1		1		1
PA1901	*phzC2*		1		1		1		1
PA1902	*phzD2*		1		1		1		1
PA1903	*phzE2*		0.998		1		0.998		1
PA1904	*phzF2*		1		1		1		1
PA1905	*phzG2*		1		1		1		1
PA4209	*phzM*	0.990	0.996	0.99	1	0.24	1	0.99	1
PA4210	*phzA1*	0.993	1	0.9966	0.994	0.9933	1	0.9966	0.994
PA4211	*phzB1*		0.991		0.997		0.997		0.997
PA4212	*phzC1*		1		1		1		1
PA4213	*phzD1*		1		1		1		1
PA4214	*phzE1*		0.998		1		0.998		1
PA4215	*phzF1*		1		1		1		1
PA4216	*phzG1*		1		1		1		1
PA4217	*phzS*	0.975	0.994	0.9957	0.999	0.9957	0.997	0.9957	0.999
PA1148	*toxA*	0.976	1	0.9558	0.996	0.7269	0.99	0.9558	0.996
PA2193	*hcnA*	0.990	1	0.9898	1	0	0	0.9898	1
PA2194	*hcnB*		0.998		0.998		0		0.998
PA2195	*hcnC*		1		0.995		0		0.995
PA1249	*aprA*	0.987	0.999	0.9933	1	0.17	1	0.9933	1
PA2570	*LecA*	0.973	1	0.9566	1	0.9766	1	0.9533	1

Table S1. Similarities of promoter and protein sequence of each quorum sensing (QS) gene in 4 different clinical isolates. The promoter and protein sequences of each of the 45 QS genes present in the four clinical isolates were compared with the same sequences present in the PAO1 strain. The blastN or blastP algorithm was used for comparison of promoter region and protein sequences, respectively. The closer the number is to 1, the closer to that of PAO1. Red box was marked because it differs significantly from that of PAO1.

PA number	gene	Transcript level of QS-related genes				
		PAO1	Y31	Y71	Y82	Y89
PA1430	*lasR*	580.2706	745.7507	577.4304	403.0809	645.7129
PA1432	*lasI*	970.7832	642.1861	680.6175	65.68094	820.1077
PA3476	*rhlI*	296.5531	483.8374	53.91151	13.68676	57.01128
PA3477	*rhlR*	171.2617	219.8335	84.06021	43.73559	84.68706
PA0996	*pqsA*	348.22	1447.974	7.276212	10.15844	7.178276
PA0997	*pqsB*	138.091	616.0806	2.885924	4.071132	3.194955
PA0998	*pqsC*	113.0327	509.6213	5.802549	5.932221	5.767517
PA0999	*pqsD*	186.9817	833.1802	8.565668	9.189127	8.547543
PA1000	*pqsE*	160.9139	796.8897	10.65336	8.995264	8.423064
PA1001	*phnA*	181.6455	798.1763	23.54791	16.47839	22.32319
PA1002	*phnB*	70.12714	175.0198	119.5509	14.07449	102.4875
PA1003	*mvfR*	159.9404	264.4327	100.5468	41.95205	98.5457
PA2587	*pqsH*	107.8047	158.2415	48.72299	14.11326	54.06528
PA2302	*ambE*	159.2553	416.4025	51.54751	22.9922	48.04881
PA2303	*ambD*	20.44323	38.54189	10.19284	1.977407	9.792331
PA2304	*ambC*	21.30856	36.23688	11.11388	3.60586	10.99563
PA2305	*ambB*	117.1069	182.5245	66.77537	25.9777	73.06904
PA2306	*ambA*	24.44535	39.61399	30.05659	28.14897	37.09468
PA1871	*lasA*	31.25976	41.16853	26.18822	23.37993	28.92057
PA3724	*lasB*	42.58106	66.95246	49.52123	55.13476	47.17746
PA3478	*rhlB*	37.49729	25.83754	25.5435	22.25552	22.77962
PA3479	*rhlA*	27.11343	38.48828	6.416575	4.846586	6.09946
PA0051	*phzH*	13.88121	27.82092	11.23668	18.18439	10.91264
PA1899	*phzA2*	1.478259	2.573033	0.491221	0.852999	0.871351
PA1900	*phzB2*	3.893949	5.038856	2.394703	2.830406	2.945998
PA1901	*phzC2*	39.11977	7.129445	3.530652	3.567087	3.319434
PA1902	*phzD2*	3.930004	3.001872	1.135949	1.512135	1.327774
PA1903	*phzE2*	15.50369	15.81343	8.043745	8.64631	6.680361
PA1904	*phzF2*	5.372208	6.432582	2.394703	2.752861	2.157632
PA1905	*phzG2*	10.70836	7.18305	5.526237	4.61395	5.601545
PA4209	*phzM*	21.05617	19.19054	12.40333	11.47672	11.78399
PA4210	*phzA1*	3.208903	6.110953	0.644728	1.046863	0.456422
PA4211	*phzB1*	6.489916	9.220035	0.890338	1.395817	0.954337
PA4212	*phzC1*	40.02115	8.630381	3.991171	4.536405	3.360927
PA4213	*phzD1*	3.641564	3.484315	1.412261	1.667226	1.410759
PA4214	*phzE1*	15.72002	14.41971	7.552524	8.336128	8.174106
PA4215	*phzF1*	5.155877	5.038856	2.824521	2.636543	2.448083
PA4216	*phzG1*	10.34781	8.469567	5.986757	4.458859	5.560052
PA4217	*phzS*	26.78893	15.27738	16.79362	13.95817	24.35635
PA1148	*toxA*	32.41352	29.16104	17.62256	25.90016	17.84196
PA2193	*hcnA*	28.51957	145.8052	3.530652	0	3.775856
PA2194	*hcnB*	98.46644	385.3653	16.57871	0.077545	15.39388
PA2195	*hcnC*	100.7019	373.9474	14.58313	0.077545	14.60551
PA1249	*aprA*	35.87481	70.86561	14.64453	15.43153	24.39784
PA2570	*lecA*	22.7147	16.72471	24.37685	37.57074	31.03671

Table S2. RNASeq results of QS-related genes. The mRNA read counts of each of 45 QS-related genes were compared with those in PAO1. Red boxes indicate that expression levels of these genes were significantly down-regulated in the MDR strains and the green box shows the expression levels of the *lasI* gene.

Gene	PAO1	Y31	Y71	Y82	Y89	Annotation information
PA2397	29.46462	1.851362	0.028754	0	0.038979	pvdE
PA2398	54.97334	0	0	0	0	fpvA
PA2399	90.64463	0.200147	1.265157	2.08585	0.896521	pvdD
PA2400	71.44488	8.105962	1.380172	0.878253	1.520188	pvdJ
Y82_2931	0.035764	0	28.91005	28.83069	27.71609	siderophore-interacting protein
Y82_2932	1.037148	3.092064	220.3721	229.0254	217.0824	Pyoverdine sidechain non-ribosomal peptide synthetase PvdI
Y82_2933	0.85833	0.838526	37.61287	49.3767	23.75665	Pyoverdine sidechain non-ribosomal peptide synthetase PvdJ
Y82_2934	3.075681	4.507077	214.7093	218.7892	215.1431	Pyoverdine sidechain non-ribosomal peptide synthetase PvdD
Y82_2935	0.107291	0.209631	106.997	197.0281	120.1973	FpvAII
Y82_2936	0.107291	0.052408	65.53939	64.65733	69.57304	pyoverdine biosynthesis protein PvdE

Table S3. Expression levels of pyoverdine-related genes in PAO1 and MDR strains. MDR strains displayed negligible expression of PAO1-derived pyoverdine-related genes, while PAO1 had little expression of MDR strain-derived pyoverdine-related genes (red box). The names of the representative genes were taken from the strain Y82 that has the largest genome among the MDR strains.

Strain number	Strain name
1	*Pseudomonas aeruginosa* AZPAE12145
2	*Pseudomonas aeruginosa* AZPAE12149
3	*Pseudomonas aeruginosa* AZPAE12413
4	*Pseudomonas aeruginosa* AZPAE12415
5	*Pseudomonas aeruginosa* AZPAE13756
6	*Pseudomonas aeruginosa* AZPAE13757
7	*Pseudomonas aeruginosa* AZPAE13850
8	*Pseudomonas aeruginosa* AZPAE13853
9	*Pseudomonas aeruginosa* AZPAE13858
10	*Pseudomonas aeruginosa* AZPAE13860
11	*Pseudomonas aeruginosa* AZPAE13872
12	*Pseudomonas aeruginosa* AZPAE13877
13	*Pseudomonas aeruginosa* AZPAE13880
14	*Pseudomonas aeruginosa* AZPAE14381
15	*Pseudomonas aeruginosa* AZPAE14442
16	*Pseudomonas aeruginosa* AZPAE14453
17	*Pseudomonas aeruginosa* AZPAE14463
18	*Pseudomonas aeruginosa* AZPAE14687
19	*Pseudomonas aeruginosa* AZPAE14688
20	*Pseudomonas aeruginosa* AZPAE14689
21	*Pseudomonas aeruginosa* AZPAE14692
22	*Pseudomonas aeruginosa* AZPAE14694
23	*Pseudomonas aeruginosa* AZPAE14700
24	*Pseudomonas aeruginosa* AZPAE14706
25	*Pseudomonas aeruginosa* AZPAE14707
26	*Pseudomonas aeruginosa* AZPAE14712
27	*Pseudomonas aeruginosa* AZPAE14713
28	*Pseudomonas aeruginosa* AZPAE14714
29	*Pseudomonas aeruginosa* AZPAE14716
30	*Pseudomonas aeruginosa* AZPAE14720
31	*Pseudomonas aeruginosa* AZPAE14729
32	*Pseudomonas aeruginosa* AZPAE14730
33	*Pseudomonas aeruginosa* AZPAE14731
34	*Pseudomonas aeruginosa* AZPAE14811
35	*Pseudomonas aeruginosa* AZPAE14819
36	*Pseudomonas aeruginosa* AZPAE14821
37	*Pseudomonas aeruginosa* AZPAE14822
38	*Pseudomonas aeruginosa* AZPAE14834
39	*Pseudomonas aeruginosa* AZPAE14852
40	*Pseudomonas aeruginosa* AZPAE14853
41	*Pseudomonas aeruginosa* AZPAE14865
42	*Pseudomonas aeruginosa* AZPAE14870
43	*Pseudomonas aeruginosa* AZPAE14887
44	*Pseudomonas aeruginosa* AZPAE14912
45	*Pseudomonas aeruginosa* AZPAE14922
46	*Pseudomonas aeruginosa* AZPAE14923
47	*Pseudomonas aeruginosa* AZPAE14929
48	*Pseudomonas aeruginosa* AZPAE14951
49	*Pseudomonas aeruginosa* AZPAE14958
50	*Pseudomonas aeruginosa* AZPAE14959
51	*Pseudomonas aeruginosa* AZPAE14984
52	*Pseudomonas aeruginosa* AZPAE15002
53	*Pseudomonas aeruginosa* AZPAE15029
54	*Pseudomonas aeruginosa* AZPAE15047
55	*Pseudomonas aeruginosa* AZPAE15065
56	*Pseudomonas aeruginosa* strain AR_0054
57	*Pseudomonas aeruginosa* strain AR_0090
58	*Pseudomonas aeruginosa* strain AR_0092
59	*Pseudomonas aeruginosa* strain AR_0094
60	*Pseudomonas aeruginosa* strain AR_0100
61	*Pseudomonas aeruginosa* strain AR_0103
62	*Pseudomonas aeruginosa* strain AR_0105
63	*Pseudomonas aeruginosa* strain AR_0108
64	*Pseudomonas aeruginosa* strain MRSN12121 strain MRSN 20176
65	*Pseudomonas aeruginosa* CCBH4851
66	*Pseudomonas aeruginosa* DHS29
67	*Pseudomonas aeruginosa* NCMG1179
68	*Pseudomonas aeruginosa* PA21_ST175
69	*Pseudomonas aeruginosa* VRFPA02
70	*Pseudomonas aeruginosa* VRFPA06

Table S4. Seventy different antibiotic-resistant *P. aeruginosa* strains. Sixty-four *P. aeruginosa* isolates (1~64), which is resistant to 3 different classes of antibiotics, were downloaded from the PATRIC website (www.patricbrc.org). Six multidrug-resistant isolates (65~70) were selected from individual studies. Please refer to the main text for references.

gene	Y71	Y82	Y89	Y71AC	Y82AC	Y89AC	Fold change	Annotation
Y82_0287	37.65271	46.00312	41.87533	72.79787	49.48585	64.84136	1.490666	NADH:flavin oxidoreductase
Y82_0288	36.61011	46.18713	42.56181	60.64997	46.38183	47.8878	1.235807	Alcohol dehydrogenase
Y82_0289	22.19246	31.94457	24.67293	19.40083	29.1492	24.42599	0.925975	hypothetical protein
Y82_0292	106.2557	168.4082	109.5139	135.1193	130.7968	74.4832	0.886046	Excinuclease ABC subunit A
Y82_0296	144.8616	104.0407	159.0213	104.854	99.64958	126.1474	0.810571	5,10-methylenetetrahydromethanopterin reductase
Y82_0299	33.89935	102.4949	38.64483	27.07161	88.33955	48.73146	0.937748	LysR family transcriptional regulator
Y82_0300	54.42365	42.43328	64.16577	47.15893	36.60597	65.24311	0.925385	Short-chain dehydrogenase
Y82_0304	178.8801	59.84086	182.4828	94.91481	61.79487	185.5652	0.812611	glyoxalase
Y82_0308	77.33103	94.65602	92.63454	87.24402	112.3511	124.2994	1.223991	antibiotic biosynthesis monooxygenase
Y82_0310	115.3412	137.3101	137.7807	112.5546	155.9857	174.0754	1.133656	LysR family transcriptional regulator
Y82_0311	121.7159	169.3283	130.2295	84.73684	170.8279	114.8182	0.879198	Alcohol dehydrogenase
Y82_1091	209.5325	53.76844	211.8399	255.0761	46.77429	231.4443	1.122393	LysR family transcriptional regulator
Y82_1092	145.6361	88.95163	149.774	198.3063	73.6044	121.9693	1.024764	Phosphonate dehydrogenase
Y82_1093	103.7535	79.05176	106.8487	117.4795	68.35969	93.7267	0.965172	Phosphonate ABC transporter permease protein phnE
Y82_1094	83.05043	72.94254	90.65586	95.72069	77.52902	93.36514	1.080949	Phosphonate ABC transporter phosphate-binding periplasmic component
Y82_1095	55.10879	38.12738	55.68571	105.0928	44.84766	69.9435	1.476505	phosphonate ABC transporter ATP-binding protein
Y82_2011	659.6671	623.1398	765.7492	201.7387	931.2045	533.0731	0.813264	NAD-dependent epimerase/dehydratase
Y82_2012	678.3147	660.2736	785.9802	228.7507	954.3597	484.7033	0.785013	aminotransferase DegT
Y82_2013	613.8225	583.0251	702.593	199.53	897.7382	406.0419	0.791449	UDP-N-acetylglucosamine 2-epimerase
Y82_2014	644.5345	595.6852	721.7337	229.5565	925.9241	434.0836	0.810195	N-acetylneuraminate synthase
Y82_2015	516.8311	466.1036	600.7919	178.0399	756.6661	376.112	0.82768	acetyltransferase
Y82_2016	595.5621	508.7945	695.8493	138.7905	776.0037	395.7573	0.728001	alcohol dehydrogenase
Y82_2017	681.3829	585.3437	827.0075	97.21306	915.5774	385.9949	0.668082	Oxidoreductase
Y82_2018	714.4184	624.4279	875.4246	92.76579	867.7327	405.359	0.616843	N-Acetylneuraminate cytidylyltransferase
Y82_2019	453.0538	402.1409	568.4869	37.6973	506.8107	217.5842	0.535297	Flagellin modification protein A
Y82_2020	261.6327	248.2696	328.8243	22.20648	345.1879	124.8216	0.586861	hypothetical protein
Y82_2021	203.0684	187.6927	246.4063	18.14723	250.8544	88.14247	0.560518	O antigen flippase
Y82_2022	303.6345	255.6669	351.5186	45.15915	398.7054	162.465	0.665696	hypothetical protein
Y82_2023	354.8112	284.9249	386.2868	84.34882	405.5556	275.154	0.745654	LPS biosynthesis protein WbpG
Y82_2024	199.4342	156.9258	228.0328	50.80032	255.0287	142.4983	0.767168	Imidazole glycerol phosphate synthase subunit HisH
Y82_2025	473.5483	398.9022	570.4252	108.9133	626.5829	339.8346	0.745269	Imidazole glycerol phosphate synthase subunit HisF
Y82_2027	714.2694	588.3983	859.0299	145.8345	917.611	489.2028	0.718254	capsular biosynthesis protein
Y82_2029	590.4683	474.7522	704.9755	123.5982	623.8	356.2258	0.623447	Glycosyltransferase
Y82_2935	106.9408	196.9301	120.1342	93.81045	223.9172	85.45079	0.950881	FpvAlll
Y82_4462	644.892	828.1297	716.6053	294.9522	649.1673	811.6018	0.801836	hypothetical protein
Y82_4478	280.1016	252.2075	675.1741	199.9479	281.1453	559.6685	0.861926	hypothetical protein
Y82_4507	40.81029	98.40987	100.1051	26.59405	138.3249	85.33027	1.045645	hypothetical protein
Y82_4508	13.37505	39.7835	34.88938	9.312396	50.84162	32.98312	1.0578	hypothetical protein
Y82_4659	1052.757	967.5008	1001.697	744.544	1036.634	1002.591	0.921182	acetyltransferase
Y82_4661	115.937	90.90216	114.6019	186.6359	140.7867	195.7695	1.627646	molecular chaperone GroEL
Y82_4665	1157.88	945.1985	1197.626	318.86	794.1997	590.5224	0.516127	aac(6')-31
Y82_4666	1507.598	1133.958	1754.524	215.379	1176.136	780.7479	0.494136	OXA-1 family class D beta-lactamase
Y82_4667	1521.956	1168.111	1583.469	526.0608	1242.926	1425.425	0.747487	aadB
Y82_4705	988.5325	680.8093	1009.773	229.7356	707.5013	529.6182	0.547515	hypothetical protein
Y82_4706	172.5352	142.0576	172.024	60.59027	136.4339	112.2471	0.635554	hypothetical protein
Y82_4707	695.3537	644.8901	714.7073	410.6409	732.2621	598.2359	0.84729	DNA-dependent ATPase, SNF2 family protein
Y82_4708	236.4912	237.3025	240.4702	374.9732	362.813	422.6741	1.624694	hypothetical protein
Y82_4710	778.2254	822.6094	801.0828	1001.351	889.3538	1458.408	1.39435	ATPase
Y82_4711	1471.047	1394.741	1499.718	1140.649	1453.714	1239.217	0.878152	hypothetical protein
Y82_5265	41.19754	103.3046	114.1981	23.34069	138.7887	97.021	1.00174	hypothetical protein
Y82_5266	16.59221	34.18952	43.81363	12.35683	39.3532	41.09834	0.981109	hypothetical protein
Y82_5575	16.29433	18.87968	15.82944	27.93719	19.97987	16.2706	1.258496	hypothetical protein
Y82_5584	179.744	221.183	188.2169	62.11249	192.663	133.7403	0.659458	hypothetical protein
Y82_5602	180.5483	198.9911	180.6656	575.6076	293.8824	407.7694	2.279986	Toxin YhaV
Y82_5603	73.72662	77.43245	73.93803	321.4568	121.5561	242.8136	3.046803	Antitoxin PrlF
Y82_6113	66.87526	45.15666	62.06595	80.61789	37.10547	49.53495	0.960714	hypothetical protein
Y82_6114	89.39539	52.14913	94.16902	129.8661	56.9783	83.4019	1.146503	phage capsid protein
Y82_6116	23.02654	20.42538	25.31903	40.08509	22.97685	22.73867	1.247629	hypothetical protein
Y82_6117	72.41592	45.56149	68.97114	182.0991	61.75919	59.21696	1.62117	RNA helicase
Y82_6118	25.37983	15.01542	24.14797	76.64819	21.19293	24.38582	1.893722	hypothetical protein
Y82_6120	26.21391	19.2477	26.65161	135.8356	30.4693	27.63994	2.689449	AlpA family transcriptional regulator
Y82_6122	52.87465	44.2366	53.46475	196.0677	56.37177	65.52433	2.11165	hypothetical protein
Y82_6123	31.84394	31.17171	30.28592	60.26195	33.39492	33.06347	1.35818	hypothetical protein
Y82_6124	145.0404	148.3877	147.8761	217.8563	157.4128	215.7361	1.339225	hypothetical protein

Table S5. Expression levels of 64 ARGs before and after antibiotic cocktail (AC) treatment. Fold changes are calculated by ratios of transcript levels after AC treatment to those before the treatment. Three well-characterized antibiotic-resistant genes (Y82_4665, Y82_4666, and Y82_4667) were highlighted with red box. Fold changes of <0.5 and >2 are marked blue or red columns, respectively. The genes with fold changes between 0.5 and 2 are shown in black columns.

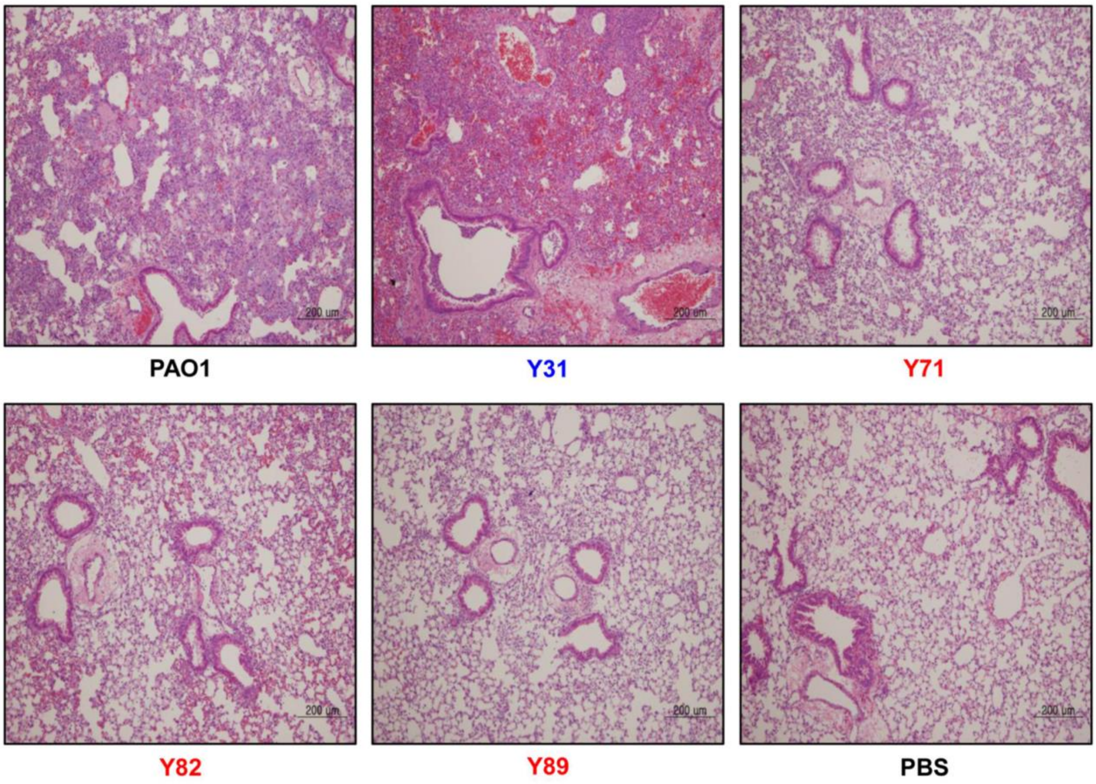

Figure S1. Histological hematoxylin/eosin (H&E) staining of mouse lung after airway infection. Eight-week-old BALB/C male mouse was infected with 2.5 × 10^7 CFU bacteria. Mouse lung of PAO1 and Y31 was extracted as soon as mouse died while that of Y71, Y82, and Y89 was extracted after 42 hours of infection. In PAO1 group, each mouse was died after infection and 14, 15, 16.5, 18, 18.5 and 19 hours. In Y31 group, each mouse was died after infection and 23, 26 and 31 hours. This mouse lung was fixed in 10 % formalin and H&E staining was performed.

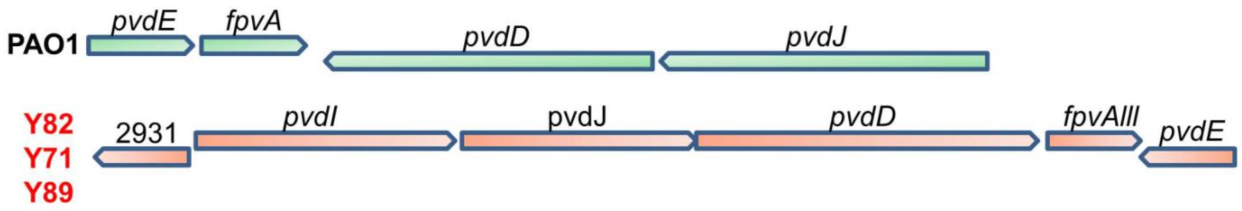

Figure S2. Arrangement of pyoverdine-related genes associated with iron acquisition. The cluster in green is extracted from PAO1 genome and the cluster in pink from MDR strains. Among the MDR strains, the genes of Y82 strain, which has the largest genome, are shown as representative genes. Y71 and Y89 also have the same arrangement as the Y82 strain.

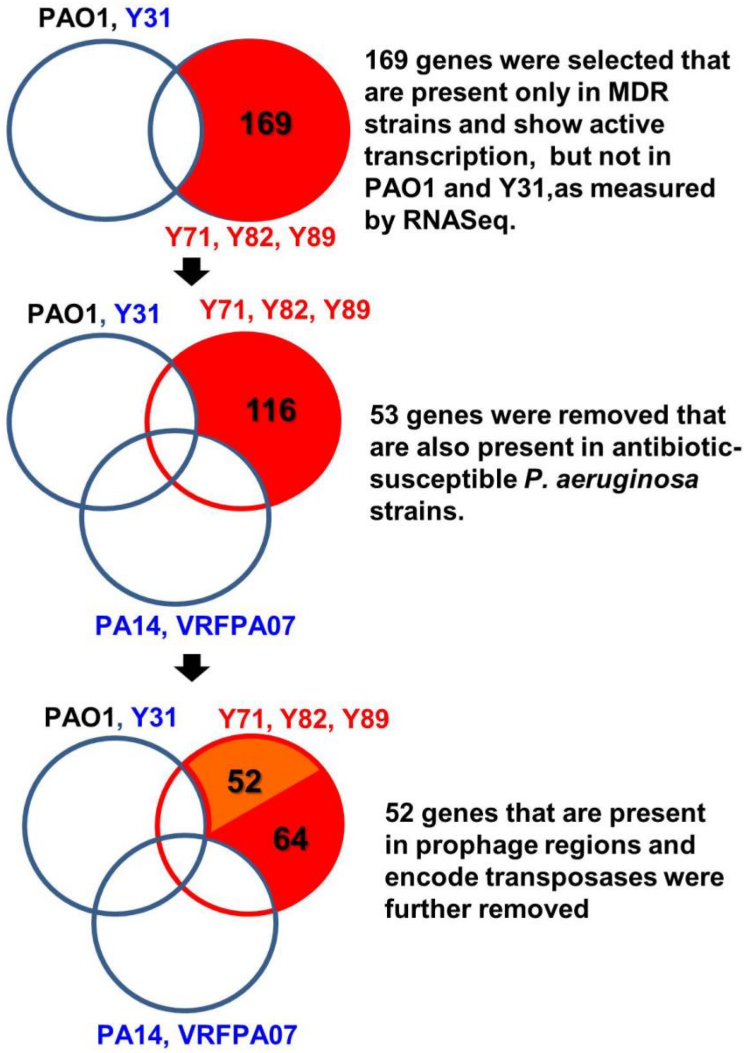

Figure S3. Selection of 64 antibiotic resistant genes (ARGs). First, 169 genes were selected that (i) were invariably present in all three MDR strains and (ii) exhibited active transcriptions as measured by RNASeq analysis. Among the 169 genes, 53 were excluded because they were found to be present in antibiotic-sensitive *P. aeruginosa* strains. Exclusion of these genes was based on clustering under both conditions (>95% sequence similarity with >95% gene length coverage (S95L95) and >50% sequence similarity with >50% gene length coverage (S50L50)) by the Blast-2.2.26 algorithm. Genes that cluster only among MDR strains under both conditions were selected as MDR strain-specific genes. Then, 52 additional genes that are present in prophage clusters and encode transposases were removed from the list, because they were not presumed to be related to antibiotic resistance.
